# Association between HEXACO personality traits and medical specialty preferences in Mexican medical students: a cross-sectional survey

**DOI:** 10.1186/s40359-020-0390-0

**Published:** 2020-03-14

**Authors:** Francisco José Barbosa-Camacho, Roberto Carlos Miranda-Ackerman, Itzel Vázquez-Reyna, Vania Brickelia Jimenez-Ley, Francisco Javier Barrera-López, Vianca Seleste Contreras-Cordero, Veronica Alexandra Sánchez-López, Tom Jilmer Castillo-Valverde, Claudina del Carmen Lamas-Abbadie, Brenda Alicia González-Adán, Ana Olivia Cortes-Flores, Gilberto Morgan-Villela, Guillermo Alonso Cervantes-Cardona, Gabino Cervantes-Guevara, Clotilde Fuentes-Orozco, Alejandro González-Ojeda

**Affiliations:** 1 Unidad de Investigación Biomédica 02 Hospital de Especialidades, Centro Médico Nacional de Occidente, Instituto Mexicano del Seguro Social, Avenida Belisario Domínguez # 1000, Col. Independencia, 44340 Guadalajara, Jalisco Mexico; 2 Hospital San Javier, Guadalajara, Jalisco Mexico; 3 ANKER Oncología Global, Guadalajara, Jalisco Mexico; 4 Hospital de Especialidades del Centro Médico Puerta de Hierro, Zapopan, Jalisco Mexico; 5 Centro Universitario de Ciencias de la Salud, Universidad de Guadalajara, Guadalajara, Jalisco Mexico; 6 Centro Universitario del Norte, Universidad de Guadalajara, Colotlán, Jalisco Mexico; 7 Centro Universitario de Tonalá, Universidad de Guadalajara, Tonalá, Jalisco Mexico

**Keywords:** Personality traits, Medical specialties, Specialty preference, Medical students, Medical career

## Abstract

**Background:**

Medical specialty is a critical choice in a physician’s life because it determines their professional future and medical practice. While some are motivated to choose a specific specialty based on the monetary gain it can provide, others are inspired by seeing the work performed by a physician or by a patient’s recovery. It is common to stereotype doctors’ personalities by their specialty.

**Methods:**

This was a cross-sectional survey study in which we administered the 100-item HEXACO Personality Inventory-Revised to 292 medical students between September 2018 and March 2019. We evaluated six different domains of personality traits. We also included questions about their medical specialty of choice, their least preferred specialty, and the motivation behind these choices. The participants included 175 women (59.9%) and 117 men (40.1%).

**Results:**

When the participants were asked about their preferred type of medical specialty, 52.4% indicated a preference for surgical specialties (surgical group) vs 47.6% who preferred clinical specialties (clinical group). We found that the surgical group showed significantly higher scores for Extraversion and Organization domains, while the clinical group showed significantly higher scores on the Honesty–Humility, Emotionality, and Agreeableness domains. We identified critical differences within the overall group of medical students by their medical specialty preference.

**Conclusions:**

Some classical stereotypes were confirmed by our results, such as surgical specialists tending to be more extroverted and organized, whereas clinical specialists were prone to being more introverted, anxious, and more emotionally attached to their patients.

## Background

The choice of a medical specialty is critical in a physician’s life because it determines their professional future and medical practice. Each specialty requires at least three, and in some cases six, years of specialized training after obtaining a medical specialty degree, which means that for a medical student to make the best of their career, it is crucial to make the correct decision about their specialty.

To many physicians, being a specialist represents not only a career choice but also, from a social standpoint, an enhancement in their quality of life and the availability of new employment opportunities. Some health-care professionals are motivated to choose a specific specialty based on the monetary gains it can provide, while others are inspired by seeing the work performed by another physician or by a patient’s recovery [1]. It is thought that many medical students would choose a high-paying specialty because of their loan debt or their goals for their future lifestyle. However, in recent years, medical residents appear to prefer a specialty involving a controlled lifestyle rather than to train for a medical specialty that has a busy or uncontrolled schedule, such as surgery or gynecology [1]. Guraya and Almaramhy [2] explored the possibility that the selection of a certain medical specialty could be influenced by whether the specialty was involved in an innovative field in current medicine or had research potential. However, it seems that the primary factor determining the motivation behind a student’s choice is their personal interest in the relevant medical field.

It is common to stereotype doctors by their specialty, where specialties are primarily classified as surgical and clinical. Surgeons are often seen as confident, practical, dynamic, less anxious, arrogant, and prone to impulsive and aggressive behavior, whereas hospital-based specialists are seen as intellectual, calm, analytic, slow at making decisions, less sociable, and more anxious [3–6]. The media usually reinforce these stereotypes, such as the medical characters of television series (e.g., *Dr. House*, *Grey’s Anatomy*, or *Scrubs*). A quick Google search for “medical specialty stereotypes” yields some common examples. Although this association between specialty choice and personality traits is often exaggerated, it seems to be consistent with the behavior of some medical students and specialists.

The five-factor model (FFM) theory of personality is usually used to explore personality differences and traits. The FFM suggests that the human personality and behavior could be explained by the exploration of five factors: Openness to Experience/intellect, Conscientiousness, Extraversion, Agreeableness, and Neuroticism (also known as emotional stability) [7, 8]. Over the years, several measurement tools have been proposed for personality studies using the FFM, such as the Zuckerman–Kuhlman personality questionnaire [5, 9], the Eysenck Personality Questionnaire (Revised) [10], the Big Five Inventory [11–13], and finally the NEO Personality Inventory [14–16]⁠. These inventories analyze the five factors and their underlying facets and provide an interpretation of human nature.

Similarly, the HEXACO Personality Inventory is a personality analysis model first proposed by Ashton and Lee in the early 2000s [17–19]. This model proposes the evaluation of six domains of personality and their underlying facets in better understanding the human personality. Each personality domain analyzes four different personality traits (also called facets), resulting in a total of 24 personality facets, in addition to “Altruism” as an interstitial scale. These six domains are: Emotionality, Extraversion, Agreeableness, Conscientiousness, Openness to experience, and Honesty–Humility. Emotionality assesses the person’s tendency to feel anxious, fearful of physical danger, and their need for emotional support from other people. Extraversion assesses the person’s confidence and response to social interactions. Agreeableness assesses the person’s response toward other people in terms of forgiveness, cooperation, and temper control. Conscientiousness assesses the person’s tendency to be organized in their environment and schedule, and their workplace diligence. Openness to experience assesses the person’s appreciation of art and nature, their creativity, and their curiosity. Finally, the HEXACO Personality Inventory’s most significant difference is the addition of the Honesty–Humility domain, which assesses the person’s tendency to manipulate or lie to others to obtain benefits, modesty, and wealth or social status. Anglim and O’Connor [20] observed that when compared to the FFM, the HEXACO Personality Inventory brings a new interpretation of the previously mentioned five domains and organizes them into six factors. The Honesty–Humility factor could be addressed as a deconstruction of the Neuroticism and Agreeableness domains of the FFM to provide a different and broader approach of these characteristics and offer a new exploration for studies in the personality field [20].

Few studies have assessed medical personnel or medical students’ personality traits and their implications towards their preference for medical specialties [5, 9–12, 21]. No study has explored the relationship between the medical students’ personality traits and their medical specialty preference using the HEXACO model. In the present study, we aimed to identify if there are any associations between the HEXACO Personality Inventory-R (HEXACO-PI-R) domains and facets and the medical students’ preferred specialty.

## Methods

### Aims

This study aimed to identify any associations between medical students’ HEXACO-PI-R personality traits and their medical specialty preference. A secondary objective was to attempt to describe the motivation behind their choice of a medical specialty.

### Design

This was a cross-sectional survey study that evaluated different personality traits using the 100-item HEXACO-PI-R (self-report form) in a sample of medical students.

### Sample

The required sample size was calculated using an infinite population formula, with an α error of 0.05 and β of 0.20, to obtain a required sample size of 222 medical students. The survey was applied to 292 medical students, for whom demographic information is outlined in Table 1. The survey was conducted between September 2018 and March 2019. We prepared an online form of the survey and distributed it to medical students via medical conferences, as well as through the authors’ students and acquaintances. All survey forms were completed fully, and we did not exclude any participants.

**Table 1:** Participants’ demographic information

Demographic Characteristics	n (%)
**Age** (years), (mean ± SD)	23.19 ± 4.5
**Sex**, n (%)	
Female	175 (59.9%)
Male	117 (40.1%)
**Semester**, n (%)	
1° Semester	3 (1.0%)
4° Semester	24 (8.2%)
5° Semester	114 (38.9%)
6° Semester	2 (0.7%)
7° Semester	6 (2.0%)
8° Semester	7 (2.4%)
9° Semester	34 (11.6%)
11° Semester	102 (35%)

### Instrument

We used the authorized Spanish translation of the 100-item HEXACO-PI-R (self-report form) [17, 18]. This instrument measures six different personality domains, each with four underlying facets: *Honesty–humility* (sincerity, fairness, greed avoidance, and modesty); *Emotionality* (fearfulness, anxiety, dependence, and sentimentality); *Extraversion* (social self-esteem, social boldness, sociability, and liveliness); *Agreeableness* (forgiveness, gentleness, flexibility, and patience); *Conscientiousness* (organization, diligence, perfectionism, and prudence); and *Openness to experience* (aesthetic appreciation, inquisitiveness, creativity, and unconventionality). In addition to these six domains, *Altruism* was measured using an interstitial scale.

In our survey, we included questions about the participants’ medical specialty of choice. In total, 42 medical specialties were categorized into clinical (CG) or surgical (SG) groups. These specialties correspond to the main specialties available (and their respective subspecialties) within the medical residence training that students can apply to undertake after obtaining their medical degree. The survey also included a four-item question asking about the motivation for their choice (e.g., family influence, other specialist’s influence, monetary motivation, or personal interest). The internal reliabilities of the domains within the study were as follows: Honesty–humility, α = 0.81; Emotionality, α = 0.82; Extraversion, α = 0.81; Agreeableness, α = 0.81; Conscientiousness, α = 0.81; and Openness to experience, α = 0.81.

### Data analysis

The data were analyzed using SPSS software (version 23.0 for Windows; IBM SPSS, Armonk, NY, USA). Descriptive analyses included proportions, means, and standard deviations. Inferential analysis of categorical variables was performed using the chi-squared test or Fisher’s exact probability test or analysis of variance, as appropriate. Student’s *t*-test was used to analyze continuous variables. To evaluate the overall effect of the inventory, a multivariate analysis of variance (MANOVA) test was performed for each facet. Multiple linear regression was used to define the likelihood of an association between a personality domain or facet and the participant’s preferred specialty or their gender. The HEXACO-PI-R domains were analyzed using exploratory (EFA) and confirmatory factor analyses (CFA) to determine the adequacy of the model within the sample. A probability level of *p* < .05 was considered significant.

## Results

### Specialty choice

We asked the medical students about their preferred type of medical specialty and found that 139 students (47.6%) preferred clinical specialties, while 153 students (52.4%) preferred surgical specialties, with a similar distribution in the sample. When comparing this preference by gender, 92 female (52.5%) and 61 male students (52.1%) preferred surgical specialties, whereas 83 female (47.4%) and 56 male students (47.8%) preferred clinical specialties. These differences were not significant using the *χ*^2^ test (*p* = .942).

Classifying the students by stage of study, 75 students (53.1%) of those attending the first half of medical school (first three years of training) and 64 students (42.3%) attending the second half of medical school preferred clinical specialties. In contrast, 66 students (46.8%) attending the first half of medical school and 87 students (57.6%) attending the second half preferred surgical specialties. This difference was not significant using the *χ*^2^ test (*p* = .079).

Afterwards, we asked about the students’ motivation for choosing a medical specialty. The overwhelming motivation for 271 students (92.8%) was personal interest in the specialty; this was followed by monetary considerations for 11 students (3.8%), the influence of another specialist for six (2%), and family influence for four students (1.4%).

When the information was stratified by type of specialty preference, 137 students (89.5%) from the SG stated that they had selected their specialty type based on personal interest, eight (5.2%) due to monetary considerations, six (3.9%) had been influenced by another specialist, and two students (1.3%) by family members. Likewise, 134 students (96.4%) from the CG answered they have selected their specialty type based on personal interest, three (2.1%) from a monetary motivation, and two (1.4%) because of family influence.

### Factorial analysis

An EFA was conducted for the six factors using maximum likelihood extraction and promax rotation. Each of the domains’ factor loadings were found to have a moderate fit with a six-factor model, and a poor fit when measuring with Harman’s single-factor test. The six-factor model loadings, average variance extracted (AVE), Composite Reliability (CR) Index, and factor variance percentages are shown in Table 2.

**Table 2:** Six-factor exploratory factor analysis models for HEXACO-PI-R

Six-Factor Model	Factor Loadings	AVE	CR Index	Factor % Variance
*Honesty–Humility*	.461–.645	.344	.673	4.43%
*Emotionality*	.527–.670	.326	.656	6.04%
*Extraversion*	.580–.850	.487	.787	15.59%
*Agreeableness*	.499–.705	.385	.711	9.10%
*Conscientiousness*	.583–.681	.400	.726	6.67%
*Openness to Experience*	.360–.649	.297	.618	3.08%

A CFA consisting of a six-factor correlated model and a one-factor model was conducted on the 24 facets of the HEXACO Personality Inventory. The fit indices of the six-factor model were: 2(237) = 629.622, *p* < .001, root mean square error of approximation (RMSEA) = .065, standardized root mean squared residual (SRMR) = .085, confirmatory fit index (CFI) = .779, and Akaike’s information criterion (AIC) =755.622. This model provides a better fit compared to the one-factor model. The comparison of the one- and six-factor models can be found in Table 3. The complete factorial analysis can be found in Additional Files 1 and 2.

**Table 3:** Comparison of confirmatory factor analysis models for HEXACO-PI-R

Models	***χ***^**2**^	***df***	***p***	CFI	RMSEA	SRMR	AIC
One-factor model	1366.49	252	< .001	.37	.12	.12	1462.49
Six-factor model	629.62	237	< .001	.78	.06	.08	755.622

Notes: *CFI* Confirmatory fit index; *RMSEA* Root mean square error of approximation; *SRMR* Standardized root mean squared residual; *AIC* Akaike’s information criterion

### Personality differences

#### Clinical vs surgical groups

A single-sample *t*-test was conducted to compare the HEXACO Personality Inventory’s scales and facets for the CG and SG’s preferences for medical specialties. The test identified that the CG had significantly higher scores for the Honesty–Humility domain (t (290) = 2.22, *p* < .05), finding significant differences in the Greed avoidance (t (290) = 2.39, *p* < .05) and Modesty (t (290) = 3.24, *p* < .001) facets. The CG also had a higher overall score for the Emotionality domain, with a significantly higher score for the Fearfulness facet (t (290) = 2.20, *p* < .05). The CG showed significantly higher overall scores for the Agreeableness domain (t (290) = 1.98, *p* < .05) and for the Flexibility facet (t (290) = 2.68, *p* < .01).

For the SG, higher overall scores were found in the Extraversion domain (t (290) = − 3.02, *p* < .001) and the Social self-esteem (t (290) = − 2.62, p < .01) and Social boldness (t (290) = − 2.95, *p* < .01) facets. Additionally, the SG presented higher overall scores for the Conscientiousness domain, with the mean score difference being significant for the Organization facet (t (290) = 2.89, *p* < .01). Both groups had similar results for both the Openness to experience and Altruism scales. The differences between the specialty preference groups are shown in Table 4.

**Table 4:** Differences in HEXACO mean scores by specialty type

HEXACO-PI-R Domains	Specialty Preference		***t*** Value †
	Mean Scores for Clinical Group (± SD)	Mean Scores for Surgical Group (± SD)	
*Honesty–Humility*	3.58 (± 0.58)	3.42 (± 0.59)	2.22 **
Sincerity	3.49 (± 0.89)	3.47 (± 0.89)	.14
Fairness	4.09 (± 0.84)	4.02 (± 0.82)	.73
Greed avoidance	2.99 (± 0.81)	2.75 (± 0.85)	2.39 *
Modesty	3.74 (± 0.76)	3.44 (± 0.78)	3.24 ***
*Emotionality*	3.15 (± 0.54)	3.05 (± 0.55)	1.64
Fearfulness	2.98 (± 0.74)	2.79 (± 0.72)	2.20 *
Anxiety	3.58 (± 0.76)	3.51 (± 0.77)	.71
Dependence	2.79 (± 0.88)	2.76 (± 0.89)	.24
Sentimentality	3.27 (± 0.79)	3.12 (± 0.76)	1.59
*Extraversion*	3.18 (± 0.63)	3.39 (± 0.56)	−3.02 **
Social self-esteem	3.46 (± 0.74)	3.68 (± 0.71)	−2.62 **
Social boldness	2.94 (± 0.92)	3.25 (± 0.85)	−2.95 **
Sociability	3.00 (± 0.78)	3.16 (± 0.68)	−1.82
Liveliness	3.30 (± 0.83)	3.46 (± 0.77)	−1.73
*Agreeableness*	2.97 (± 0.59)	2.84 (± 0.55)	1.98 *
Forgiveness	2.94 (± 0.79)	2.91 (± 0.81)	.31
Gentleness	3.09 (± 0.69)	2.95 (± 0.65)	1.79
Flexibility	2.91 (± 0.73)	2.68 (± 0.74)	2.68 **
Patience	2.95 (± 0.86)	2.82 (± 0.83)	1.31
*Conscientiousness*	3.69 (± 0.55)	3.78 (± 0.53)	−1.42
Organization	3.45 (± 0.86)	3.74 (± 0.86)	−2.89 **
Diligence	4.08 (± 0.65)	4.20 (± 0.57)	−1.60
Perfectionism	3.77 (± 0.69)	3.75 (± 0.66)	.21
Prudence	3.45 (± 0.73)	3.43 (± 0.79)	.30
*Openness to experience*	3.74 (± 0.49)	3.77 (± 0.46)	−.55
Aesthetic appreciation	4.11 (± 0.69)	4.11 (± 0.63)	.07
Inquisitiveness	3.76 (± 0.73)	3.81 (± 0.71)	−.51
Creativity	3.70 (± 0.74)	3.78 (± 0.72)	−.98
Unconventionality	3.37 (± 0.61)	3.38 (± 0.63)	−.17
*Altruism*	3.66 (± 0.68)	3.65 (± 0.70)	.61

Notes: † *t* values obtained by Student’s *t* test (*p < .05, **p < .01, ***p < .001)

Univariate analysis degrees of freedom: 290

To determine any overall significant associations, we performed a MANOVA test including the univariate *t* test for statistically significant factors and covaried by gender. The Hotelling’s Trace analysis indicated statistical significance between both groups (T^2^ = .125, multivariate F (10, 280) = 222.59, *p* < .001). These differences remained statistically significant for the Honesty–Humility (t (2) = 7.21, *p* < .001) and Extraversion scales (t (2) = 4.85, *p* < .01), as well as the Greed avoidance (t (2) = 3.89, *p* < .05), Modesty (t (2) = 13.94, *p* < .001), Fearfulness (t (2) = 9.97, *p* = .001), Social self-esteem (t (2) = 3.51, *p* < .05), Social boldness (t (2) = 7.09, *p* < .001), Flexibility (t (2) = 3.71, *p* < .05), and Organization facets (t (2) = 4.18, *p* < .05).

A multiple linear regression model was constructed by entering the six main HEXACO-PI-R domains and the Altruism scale. The forward model used a probability F score of .05 to enter and .10 to be eliminated. A significant regression equation was found (F (5, 286) = 7.881, *p* < .001), with an R^2^ of .121. On the one hand, a higher score in the Modesty (β = −.91, t (291) = − 2.41, *p* < .05), Flexibility (β = −.11, t (291) = − 2.84, *p* < .01), and Perfectionism facets (β = −.09, t (291) = − 2.02, *p* < .05) were found to be predictors for preferring a clinical medical specialty. On the other hand, participants with higher scores in the Extraversion scale (β = .13, t (291) = 2.74, *p* < .01), and organization facet (β = .12, t (291) = 3.60, *p* < .01) were found to be predictors for preferring a surgical medical specialty.

#### Gender differences

As a secondary analysis, we compared men and women within the medical student sample to identify differences between genders. When we compared the HEXACO mean scores for men and women using Student’s t test, we found that female students had significantly higher scores for the Honesty–Humility domain (t (290) = − 2.93, *p* < .01), and the Fairness (t (290) = − 2.55, *p* < .01) and Modesty (t (290) = − 3.86, *p* < .001) facets. Similarly, female students had significantly higher scores for the overall Emotionality domain (t (290) = − 5.22, *p* < .001) and all the facets within it: Fearfulness (t (290) = − 3.88, *p* < .001), Anxiety (t (290) = − 3.44, *p* < .001), Dependence (t (290) = − 3.02, *p* < .01), and Sentimentality (t (290) = − 3.86, *p* < .001). Additionally, in the Openness to experience domain, female students scored higher in the Aesthetic appreciation facet (t (290) = − 2.85, *p* < .01). Finally, female students showed significantly higher scores than male students on the Altruism scale (t (290) = − 3.28, *p* < .001).

Male students showed overall higher scores for the Extraversion domain, with a significant difference in the Social boldness facet (t (290) = 2.24, *p* < .05). In addition, male students had higher overall scores for the Agreeableness and Conscientiousness domains than did female students, but these differences did not reach statistical significance. For the Openness to experience domain, the male students had significantly higher scores in the Inquisitiveness facet (t (290) = 2.48, *p* < .05). The complete mean score differences between genders are presented in Table 5.

**Table 5:** Gender differences in medical students

HEXACO-PI-R Domains	Gender		***t*** Values †
	Mean Scores for Male Students (± SD)	Mean Scores for Female Students (± SD)	
*Honesty–Humility*	3.37 (± 0.64)	3.58 (± 0.55)	−2.93 **
Sincerity	3.43 (± 0.93)	3.51 (± 0.86)	−.75
Fairness	3.90 (± 0.89)	4.16 (± 0.78)	−2.55 **
Greed avoidance	2.78 (± 0.92)	2.92 (± 0.78)	−1.37
Modesty	3.36 (± 0.85)	3.73 (± 0.70)	−3.86 ***
*Emotionality*	2.90 (± 0.50)	3.23 (± 0.54)	−5.22 ***
Fearfulness	2.69 (± 0.68)	3.02 (± 0.74)	−3.88 ***
Anxiety	3.36 (± 0.75)	3.67 (± 0.75)	−3.44 ***
Dependence	2.59 (± 0.82)	2.90 (± 0.91)	−3.02 **
Sentimentality	2.98 (± 0.76)	3.34 (± 0.76)	−3.86 ***
*Extraversion*	3.32 (± 0.60)	3.27 (± 0.60)	.67
Social self-esteem	3.59 (± 0.64)	3.57 (± 0.78)	.33
Social boldness	3.25 (± 0.90)	3.01 (± 0.88)	2.24 *
Sociability	3.07 (± 0.81)	3.10 (± 0.68)	−.32
Liveliness	3.36 (± 0.80)	3.40 (± 0.80)	−.45
*Agreeableness*	2.91 (± 0.54)	2.90 (± 0.59)	.20
Forgiveness	2.92 (± 0.80)	2.93 (± 0.80)	−.13
Gentleness	2.97 (± 0.68)	3.05 (± 0.67)	−1.02
Flexibility	2.76 (± 0.71)	2.81 (± 0.77)	−.51
Patience	3.00 (± 0.86)	2.80 (± 0.82)	1.92
*Conscientiousness*	3.74 (± 0.59)	3.73 (± 0.51)	.10
Organization	3.60 (± 0.82)	3.60 (± 0.91)	.06
Diligence	4.15 (± 0.66)	4.14 (± 0.58)	.14
Perfectionism	3.68 (± 0.73)	3.82 (± 0.63)	−1.69
Prudence	3.53 (± 0.79)	3.38 (± 0.74)	1.65
*Openness to experience*	3.77 (± 0.46)	3.74 (± 0.48)	.43
Aesthetic appreciation	3.98 (± 0.67)	4.20 (± 0.63)	−2.85 **
Inquisitiveness	3.91 (± 0.63)	3.70 (± 0.76)	2.48 *
Creativity	3.75 (± 0.69)	3.73 (± 0.76)	.20
Unconventionality	3.44 (± 0.63)	3.34 (± 0.61)	1.33
*Altruism*	3.49 (± 0.74)	3.75 (± 0.64)	−3.28 ***

Notes: † *t* values obtained by Student’s *t* test (*p < .05, **p < .01, ***p < .001)

Univariate analysis degrees of freedom: 290

A multiple linear regression model was constructed by entering the six main HEXACO scales and the Altruism scale. The forward model used a probability F score of .05 to enter and .10 to be eliminated. A significant regression equation was found (F (4, 287) = 15.85, *p* < .001), with an R^2^ of .181. Being a woman was a positive predictor to scoring higher in the Emotionality domain (β = .21, t (291) = 2.41, *p* < .01) Modesty (β = .11, t (291) = 3.28, *p* < .001), and Aesthetic appreciation facets (β = .17, t (291) = 3.97, *p* < .001). Meanwhile, being male seems to be a predictor for a higher Inquisitiveness facet score (β = −.15, t (291) = − 3.85, *p* < .001).

## Discussion

During their training, medical students experience what it is like to practice different medical specialties ranging from hospital-based specialties such as internal medicine, pathology, or psychiatry, to surgical specialties such as general surgery, gynecology, or traumatology. Although certain personality characteristics can be associated with all medical specialties, physicians can present outlying personality traits and are not bound to a “default” personality type. In the present study, we aimed to evaluate whether there was a relationship between students’ expected personality traits and their selection of medical specialties.

The primary objective of our study was to evaluate any association between personality characteristics and preference for a particular type of specialty. Previous studies have suggested that surgeons tend to be more confident, bolder, and less anxious than clinical specialists [3, 5, 22]. The students in our study who preferred surgical specialties could be described as less modest, more extroverted, more diligent, more organized, and overall less emotional, than their clinical counterparts. Hughes et al. [11] analyzed the association of Big 5 scores with performance in surgical specialties, finding that higher scores for the extraversion, conscientiousness, and emotional stability scales could predict better performance during surgical training. These are the domains where we found that the SG scored higher, suggesting that these traits not only support the past studies theory but could also be a prominent factor in their performance during their professional practice because a more-organized and less-emotional surgeon could make better emergency and surgical decisions in the operating room compared to a more anxious surgeon.

In contrast, we found that students in the CG seemed to be more patient, gentle, and have a stronger overall emotional response, which could translate to a stronger emotional bond between the patient and physician that is often found in clinical specialists compared with surgical specialists [4, 5, 12, 23].

The multiple regression analysis identified significant differences between the CG and SG, indicating that having higher mean scores for the Extraversion domain could increase the likelihood that a student would prefer a surgical specialty, whereas having higher mean scores for the Emotionality, Agreeableness, and Honesty–Humility domains could increase the likelihood of a preferring clinical specialties.

Using personality inventories such as HEXACO, vocational mentors and medical school educators could use the found associations to guide medical students to take on a medical specialty path more fitted to their personality affinities. Personalized extracurricular training, such as surgical or clinical rotations depending of the student preferences, could help future health-care professionals in their decision-making process, as the study of the theory of the different subjects cannot be compared to hands-on experience in the field. This personality evaluation could help clarify the students’ doubts about their specialty preferences, their medical practice, and finally guide them through to making the best decision about the program of their choice based on their personality traits.

Savickas [24] proposed that the practice of career interventions that enhance the sense of self-identity are more adequate path when assisting the life design of clients. Similarly, other authors [25] suggest that therapists should counsel using a constructive approach instead of guiding the students by scoring their personality using inventories or other linear approaches. Of course, the HEXACO Personality Inventory should be taken as a guiding tool because not all clinical or surgical specialists can be assumed to present the same personality types. We suggest that combining both a constructive counseling and personality inventories, an integral guidance can be achieved.

Our results indicated that medical students’ preferences are evenly distributed between clinical and surgical specialties. This equality applies to both genders and to those within the first and second halves of medical school. In contrast, previous studies reported a higher overall preference for clinical specialties [2, 5, 9, 26, 27]. Most of our participants had selected their specialty type because of personal interest, rather than from monetary motives or because of the influence of others (either specialists or family members). This finding seems to be similar to the results of previous studies [2, 12].

In the secondary analysis, we found significant differences between the personalities of men and women in our medical student sample, mainly in the Honesty–Humility, Emotionality, and Altruism scales. This finding is similar to the results of a study by Hojat and Zuckerman [5], but in contrast to those obtained by Kwon and Park [12], who found no difference between genders in their sample. However, a study by Mullola et al. [28] found that female specialists scored higher for extraversion and conscientiousness than male specialists, while Weisberg et al. [29] analyzed Big 5 scores by gender and reported that although women had a tendency to score higher for Agreeableness, Extraversion, and Neuroticism, there were no differences between the genders for the Conscientiousness and Openness to experience scales. These findings differ from our results indicating that female students had lower scores than men for Extraversion, but similar scores for Conscientiousness and Agreeableness. We can infer that based on these results, that female medical students could become more emotional, altruistic, and humble specialists when compared to male medical students. However, higher scores in extroversion and emotional stability can be associated with higher performance [11], as found mostly in our male sample. In our sample, as Mexico is a more conservative country, we expected to find a preference for clinical specialties rather than surgical specialties in our female sample because of the popular belief that women would tend to prefer more controlled and flexible scheduled specialties, in case they planned to start a family. Nonetheless, our results report a similar distribution of preferences between men and women, which “breaks” the paradigm that surgical specialties are mostly male-dominant because as both men and women in our sample aimed to achieve this goal.

### Study limitations

One limitation of our study is our sample size. Although we were able to find significant differences between specialty preferences and between genders, a larger sample size is needed to find a higher factor adequacy in the model to explore further the personality differences (as explained by the EFA and CFA results) and generalize our results to broader populations. We intended to perform an analysis of individual medical specialties (e.g., pediatrics or general surgery), but the small number of participants made it difficult to achieve an adequate sample within each specialty. In the future, we intend to survey a higher number of participants who aim to apply for each of the various specialties available and perform a personality analysis for each specialty with the aim of identifying its core characteristics.

## Conclusion

We found differences in the studied personality domains within groups of medical students stratified by gender or by medical specialty preference. Those in the SG were more likely to be extroverted and organized, whereas those in the CG were more likely to be introverted, anxious, and emotionally attached to their patients. These findings could be used by education and vocational guidance professionals to guide students toward the medical specialty in which they are likely to perform better and assist them to best plan for their future professional practice.

## Supplementary information

**Additional file 1.** HEXACO-PI-R factorial analyses. Factor analysis comparing a 6-Factor model and one-factor model of the HEXACO-PI-R domains and the underlying facets, including all items for each facet. The analysis compares the models’ AVE, CR indexes, KMO tests and variance percentage scores.

**Additional file 2.** HEXACO-PI-R factorial analyses (facet scores). Factor analysis comparing a of the 6-Factor model and the one-factor model of the HEXACO-PI-R domains and the underlying facets, including all only the mean scores for each facet. The analysis compares the models’ AVE, CR indexes, KMO tests and variance percentage scores.

## Data Availability

The data sets used and/or analyzed during the current study are available from the corresponding author on reasonable request.

## Abbreviations

CFA: Confirmatory factor analysis
CG: Clinical group
EFA: Exploratory factor analysis
FFM: Five-factor model
HEXACO-PI-R: HEXACO Personality Inventory Revised
SG: Surgical group
