# Supplementary material for: Association between HEXACO personality traits and medical specialty preferences in Mexican medical students: a cross-sectional survey
Source: BMC Psychol. 2020 Mar 14;8:23. doi: 10.1186/s40359-020-0390-0 (PMC7071694; doi:10.1186/s40359-020-0390-0)
Supplement: Supplementary file 1 — Additional file 1. HEXACO-PI-R factorial analyses. Factor analysis comparing a 6-Factor model and one-factor model of the HEXACO-PI-R domains and the underlying facets, including all items for each facet. The analysis compares the models’ AVE, CR indexes, KMO tests and variance percentage scores. [file 40359_2020_390_MOESM1_ESM.docx]

**Additional file 1.** HEXACO-PI-R factorial analyses

|  | **CFA 6-Factor Analysis** | | | | | **Harman’s Single-Factor Test** | | | | |
| --- | --- | --- | --- | --- | --- | --- | --- | --- | --- | --- |
|  | **λ** | **AVE** | **CR Indexes** | **KMO Test** | **% Variance** | **λ** | **AVE** | **CR Indexes** | **KMO Test** | **% Variance** |
| ***Honesty–humility*** |  | .243 | .831 | 0.7 | 31.88% |  | .058 | .464 | 0.7 | 8.77% |
| Sincerity item 1 | .455 |  |  |  |  | .119 |  |  |  |  |
| Sincerity item 2 | .537 |  |  |  |  | .152 |  |  |  |  |
| Sincerity item 3 | .589 |  |  |  |  | .191 |  |  |  |  |
| Sincerity item 4 | .535 |  |  |  |  | .141 |  |  |  |  |
| Fairness item 1 | .464 |  |  |  |  | .325 |  |  |  |  |
| Fairness item 2 | .316 |  |  |  |  | .343 |  |  |  |  |
| Fairness item 3 | .470 |  |  |  |  | .253 |  |  |  |  |
| Fairness item 4 | .464 |  |  |  |  | .327 |  |  |  |  |
| Greed avoidance item 1 | .329 |  |  |  |  | .124 |  |  |  |  |
| Greed avoidance item 2 | .557 |  |  |  |  | .146 |  |  |  |  |
| Greed avoidance item 3 | .621 |  |  |  |  | .304 |  |  |  |  |
| Greed avoidance item 4 | .623 |  |  |  |  | .352 |  |  |  |  |
| Modesty item 1 | .332 |  |  |  |  | .132 |  |  |  |  |
| Modesty item 2 | .461 |  |  |  |  | .162 |  |  |  |  |
| Modesty item 3 | .373 |  |  |  |  | .262 |  |  |  |  |
| Modesty item 4 | .592 |  |  |  |  | .281 |  |  |  |  |
| ***Emotionality*** |  | .219 | .806 |  |  |  | .026 | .102 |  |  |
| Fearfulness item 1 | .376 |  |  |  |  | .016 |  |  |  |  |
| Fearfulness item 2 | .303 |  |  |  |  | .044 |  |  |  |  |
| Fearfulness item 3 | .459 |  |  |  |  | .110 |  |  |  |  |
| Fearfulness item 4 | .479 |  |  |  |  | .257 |  |  |  |  |
| Anxiety item 1 | .505 |  |  |  |  | .280 |  |  |  |  |
| Anxiety item 2 | .413 |  |  |  |  | .035 |  |  |  |  |
| Anxiety item 3 | .162 |  |  |  |  | .144 |  |  |  |  |
| Anxiety item 4 | .463 |  |  |  |  | .355 |  |  |  |  |
| Dependence item 1 | .668 |  |  |  |  | .074 |  |  |  |  |
| Dependence item 2 | .609 |  |  |  |  | .231 |  |  |  |  |
| Dependence item 3 | .653 |  |  |  |  | .047 |  |  |  |  |
| Dependence item 4 | .380 |  |  |  |  | .010 |  |  |  |  |
| Sentimentality item 1 | .477 |  |  |  |  | .013 |  |  |  |  |
| Sentimentality item 2 | .309 |  |  |  |  | .101 |  |  |  |  |
| Sentimentality item 3 | .448 |  |  |  |  | .084 |  |  |  |  |
| Sentimentality item 4 | .500 |  |  |  |  | .175 |  |  |  |  |
| ***Extraversion*** |  | .282 | .854 |  |  |  | .149 | .714 |  |  |
| Social self-esteem item 1 | .466 |  |  |  |  | .482 |  |  |  |  |
| Social self-esteem item 2 | .502 |  |  |  |  | .407 |  |  |  |  |
| Social self-esteem item 3 | .653 |  |  |  |  | .409 |  |  |  |  |
| Social self-esteem item 4 | .375 |  |  |  |  | .499 |  |  |  |  |
| Social boldness item 1 | .576 |  |  |  |  | .257 |  |  |  |  |
| Social boldness item 2 | .636 |  |  |  |  | .298 |  |  |  |  |
| Social boldness item 3 | .536 |  |  |  |  | .213 |  |  |  |  |
| Social boldness item 4 | .468 |  |  |  |  | .356 |  |  |  |  |
| Sociability item 1 | .104 |  |  |  |  | .082 |  |  |  |  |
| Sociability item 2 | .540 |  |  |  |  | .332 |  |  |  |  |
| Sociability item 3 | .419 |  |  |  |  | .311 |  |  |  |  |
| Sociability item 4 | .465 |  |  |  |  | .222 |  |  |  |  |
| Liveliness item 1 | .548 |  |  |  |  | .405 |  |  |  |  |
| Liveliness item 2 | .574 |  |  |  |  | .539 |  |  |  |  |
| Liveliness item 3 | .604 |  |  |  |  | .582 |  |  |  |  |
| Liveliness item 4 | .733 |  |  |  |  | .437 |  |  |  |  |
| ***Agreeableness*** |  | .219 | .805 |  |  |  | .110 | .626 |  |  |
| Forgiveness item 1 | .389 |  |  |  |  | .254 |  |  |  |  |
| Forgiveness item 2 | .490 |  |  |  |  | .426 |  |  |  |  |
| Forgiveness item 3 | .156 |  |  |  |  | .237 |  |  |  |  |
| Forgiveness item 4 | .574 |  |  |  |  | .419 |  |  |  |  |
| Gentleness item 1 | .483 |  |  |  |  | .223 |  |  |  |  |
| Gentleness item 2 | .497 |  |  |  |  | .409 |  |  |  |  |
| Gentleness item 3 | .143 |  |  |  |  | -.079 |  |  |  |  |
| Gentleness item 4 | .510 |  |  |  |  | .314 |  |  |  |  |
| Flexibility item 1 | .433 |  |  |  |  | .169 |  |  |  |  |
| Flexibility item 2 | .408 |  |  |  |  | .335 |  |  |  |  |
| Flexibility item 3 | .462 |  |  |  |  | .403 |  |  |  |  |
| Flexibility item 4 | .464 |  |  |  |  | .364 |  |  |  |  |
| Patience item 1 | .664 |  |  |  |  | .372 |  |  |  |  |
| Patience item 2 | .513 |  |  |  |  | .387 |  |  |  |  |
| Patience item 3 | .585 |  |  |  |  | .437 |  |  |  |  |
| Patience item 4 | .409 |  |  |  |  | .213 |  |  |  |  |
| ***Conscientiousness*** |  | .263 | .848 |  |  |  | .126 | .678 |  |  |
| Organization item 1 | .417 |  |  |  |  | .298 |  |  |  |  |
| Organization item 2 | .556 |  |  |  |  | .337 |  |  |  |  |
| Organization item 3 | .388 |  |  |  |  | .257 |  |  |  |  |
| Organization item 4 | .672 |  |  |  |  | .473 |  |  |  |  |
| Diligence item 1 | .573 |  |  |  |  | .390 |  |  |  |  |
| Diligence item 2 | .482 |  |  |  |  | .320 |  |  |  |  |
| Diligence item 3 | .512 |  |  |  |  | .421 |  |  |  |  |
| Diligence item 4 | .519 |  |  |  |  | .494 |  |  |  |  |
| Perfectionism item 1 | .417 |  |  |  |  | .147 |  |  |  |  |
| Perfectionism item 2 | .409 |  |  |  |  | .300 |  |  |  |  |
| Perfectionism item 3 | .599 |  |  |  |  | .352 |  |  |  |  |
| Perfectionism item 4 | .545 |  |  |  |  | .109 |  |  |  |  |
| Prudence item 1 | .477 |  |  |  |  | .376 |  |  |  |  |
| Prudence item 2 | .585 |  |  |  |  | .428 |  |  |  |  |
| Prudence item 3 | .406 |  |  |  |  | .407 |  |  |  |  |
| Prudence item 4 | .552 |  |  |  |  | .324 |  |  |  |  |
| ***Openness to experience*** |  | .170 | .745 |  |  |  | .057 | .454 |  |  |
| Aesthetic appreciation item 1 | .406 |  |  |  |  | .211 |  |  |  |  |
| Aesthetic appreciation item 2 | .433 |  |  |  |  | .279 |  |  |  |  |
| Aesthetic appreciation item 3 | .477 |  |  |  |  | .204 |  |  |  |  |
| Aesthetic appreciation item 4 | .423 |  |  |  |  | .208 |  |  |  |  |
| Inquisitiveness item 1 | .550 |  |  |  |  | .284 |  |  |  |  |
| Inquisitiveness item 2 | .475 |  |  |  |  | .175 |  |  |  |  |
| Inquisitiveness item 3 | .402 |  |  |  |  | .304 |  |  |  |  |
| Inquisitiveness item 4 | .481 |  |  |  |  | .336 |  |  |  |  |
| Creativity item 1 | .071 |  |  |  |  | .329 |  |  |  |  |
| Creativity item 2 | .542 |  |  |  |  | .215 |  |  |  |  |
| Creativity item 3 | .363 |  |  |  |  | .265 |  |  |  |  |
| Creativity item 4 | .260 |  |  |  |  | .236 |  |  |  |  |
| Unconventionality item 1 | .167 |  |  |  |  | .206 |  |  |  |  |
| Unconventionality item 2 | .330 |  |  |  |  | .023 |  |  |  |  |
| Unconventionality item 3 | .290 |  |  |  |  | .013 |  |  |  |  |
| Unconventionality item 4 | .565 |  |  |  |  | .257 |  |  |  |  |
| Notes: λ denotes factor loadings. CFA, confirmatory factor analysis; KMO, Kaiser–Meyer–Olkin test; AVE, average variance extracted; CR, composite reliability. | | | | | | | | | | |
