# Supplementary material for: Association between HEXACO personality traits and medical specialty preferences in Mexican medical students: a cross-sectional survey
Source: BMC Psychol. 2020 Mar 14;8:23. doi: 10.1186/s40359-020-0390-0 (PMC7071694; doi:10.1186/s40359-020-0390-0)
Supplement: Supplementary file 2 — Additional file 2. HEXACO-PI-R factorial analyses (facet scores). Factor analysis comparing a of the 6-Factor model and the one-factor model of the HEXACO-PI-R domains and the underlying facets, including all only the mean scores for each facet. The analysis compares the models’ AVE, CR indexes, KMO tests and variance percentage scores. [file 40359_2020_390_MOESM2_ESM.docx]

**Additional file 2.** HEXACO-PI-R factorial analyses (facet scores)

|  | **CFA 6-Factor Analysis** | | | | |  | **Harman’s Single-Factor Test** | | | | |
| --- | --- | --- | --- | --- | --- | --- | --- | --- | --- | --- | --- |
|  | **λ** | **AVE** | **CR Indexes** | **Factor % Variance** | **KMO Test** | **Total % Variance** | **λ** | **AVE** | **CR Indexes** | **KMO Test** | **Total % Variance** |
| ***Honesty–humility*** |  | .344 | .673 | 4.43% | .769 | 44.94% |  | .065 | .198 | .769 | 14.74% |
| Sincerity | .583 |  |  |  |  |  | .151 |  |  |  |  |
| Fairness | .461 |  |  |  |  |  | .373 |  |  |  |  |
| Greed avoidance | .645 |  |  |  |  |  | .226 |  |  |  |  |
| Modesty | .638 |  |  |  |  |  | .214 |  |  |  |  |
| ***Emotionality*** |  | .326 | .656 | 6.04% |  |  |  | .142 | .235 |  |  |
| Fearfulness | .527 |  |  |  |  |  | -.167 |  |  |  |  |
| Anxiety | .541 |  |  |  |  |  | -.267 |  |  |  |  |
| Dependence | .670 |  |  |  |  |  | -.68 |  |  |  |  |
| Sentimentality | .534 |  |  |  |  |  | .085 |  |  |  |  |
| ***Extraversion*** |  | .487 | .787 | 15.59% |  |  |  | .325 | .643 |  |  |
| Social self-esteem | .703 |  |  |  |  |  | .699 |  |  |  |  |
| Social boldness | .628 |  |  |  |  |  | .429 |  |  |  |  |
| Sociability | .580 |  |  |  |  |  | .395 |  |  |  |  |
| Liveliness | .850 |  |  |  |  |  | .686 |  |  |  |  |
| ***Agreeableness*** |  | .385 | .711 | 9.10% |  |  |  | .150 | .404 |  |  |
| Forgiveness | .499 |  |  |  |  |  | .455 |  |  |  |  |
| Gentleness | .669 |  |  |  |  |  | .267 |  |  |  |  |
| Flexibility | .588 |  |  |  |  |  | .366 |  |  |  |  |
| Patience | .705 |  |  |  |  |  | .433 |  |  |  |  |
| ***Conscientiousness*** |  | .400 | .726 | 6.67% |  |  |  | .205 | .496 |  |  |
| Organization | .583 |  |  |  |  |  | .421 |  |  |  |  |
| Diligence | .628 |  |  |  |  |  | .562 |  |  |  |  |
| Perfectionism | .681 |  |  |  |  |  | .306 |  |  |  |  |
| Prudence | .633 |  |  |  |  |  | .481 |  |  |  |  |
| ***Openness to experience*** |  | .297 | .618 | 3.08% |  |  |  | .113 | .328 |  |  |
| Aesthetic appreciation | .649 |  |  |  |  |  | .330 |  |  |  |  |
| Inquisitiveness | .524 |  |  |  |  |  | .388 |  |  |  |  |
| Creativity | .360 |  |  |  |  |  | .382 |  |  |  |  |
| Unconventionality | .603 |  |  |  |  |  | .218 |  |  |  |  |
| Notes: λ denotes factor loadings. CFA, confirmatory factor analysis; KMO, Kaiser–Meyer–Olkin test; AVE, average variance extracted; CR, composite reliability. | | | | | | | | | | | |
